# Biomass Functions and Carbon Content Variabilities of Natural and Planted *Pinus koraiensis* in Northeast China

**DOI:** 10.3390/plants10020201

**Published:** 2021-01-21

**Authors:** Faris Rafi Almay Widagdo, Lihu Dong, Fengri Li

**Affiliations:** Key Laboratory of Sustainable Forest Ecosystem Management-Ministry of Education, School of Forestry, Northeast Forestry University, Harbin 150040, China; faris.almai@gmail.com or

**Keywords:** additive biomass equations, root-to-shoot ratio, carbon concentration, biomass partitioning, forest origin

## Abstract

The population of natural Korean pine (*Pinus koraiensis*) in northeast China has sharply declined due to massive utilization for its high-quality timber, while this is vice versa for Korean pine plantations after various intensive afforestation schemes applied by China’s central authority. Hence, more comprehensive models are needed to appropriately understand the allometric relationship variations between the two origins. In this study, we destructively sampled *Pinus koraiensis* from several natural and plantation sites in northeast China to investigate the origin’s effect on biomass equations. Nonlinear seemingly unrelated regression with weighted functions was used to present the additivity property and homogenize the model residuals in our two newly developed origin-free (population average) and origin-based (dummy variable) biomass functions. Variations in biomass allocations, carbon content, and root-to-shoot ratio between the samples obtained from plantations and natural stands were also investigated. The results showed that (1) involving the origin’s effect in dummy variable models brought significant improvement in model performances compared to the population average models; (2) incorporating tree total height (H) as an additional predictor to diameter at breast height (D) consistently increase the models’ accuracy compared to using D only as of the sole predictors for both model systems; (3) stems accounted for the highest partitioning proportions and foliage had the highest carbon content among all biomass components; (4) the root-to-shoot ratio ranged from 0.18–0.35, with plantations (0.28 ± 0.04) had slightly higher average value (±SD) compared to natural forests (0.25 ± 0.03). Our origin-based models can deliver more accurate individual tree biomass estimations for *Pinus koraiensis*, particularly for the National Forest Inventory of China.

## 1. Introduction

Terrestrial living plants hold a crucial role in regulating the climate through their contributions in carbon flux density and storing a tremendous amount of carbon storage (380–536 Pg C) [1,2]. The plant’s interaction in competing for both below- and above-ground resources (i.e., soil nutrients, water, and light) shapes the forest’s composition and structure, determining the forest community’s capacity in storing carbon [3]. However, the forest community’s carbon sequestration capacity has gained an increasing number of public concerns due to the more apparent appalling evidence of climate change and global warming [4,5]. Carbon sequestration is highly associated with forest biomass; thus, accurate biomass estimation is a key step for carbon appraisals and a primary tool for the successful implementation of climate change mitigation strategies [6].

An individual tree biomass equation is widely known to be one of the most efficient techniques to estimate forest biomass since direct biomass measurements need tremendous resources (i.e., labor, time, equipment, and cost). Thousands of equations for stem, root, crown variables (i.e., branch and foliage), aboveground, and total biomass have been developed worldwide [7,8,9,10]. Most of them used the allometric function (Y = aX^b^) to relate the required biomass (Y) with several easy-to-measure independent variables (X), in which tree diameter at breast height (D) is independently applied as the sole predictor. If several biomass component equations are developed, the inherent correlation among them is often neglected, nullifying their estimations’ additive property. As a result, the summation of each component’s prediction will yield either an over- or under-estimation compared to the total biomass prediction [11,12]. Hence, several researchers have specifically addressed the importance of considering compatibility property in establishing tree-level biomass equations [8,13,14,15,16]. Among various model specifications for nonlinear function, nonlinear seemingly unrelated regression (NSUR) emerges as a primary standard [13,17,18,19,20], owing to its flexibility, allowing each biomass component to have different combinations of predictors and weighting function to increase the model’s accuracy and homogenize the model residuals, respectively.

Besides biomass equations, an accurate carbon content value has also been recognized as an important component since it will be multiplied by the biomass estimation result for carbon stock quantification. In recent decades, there has been a slow but growing trend in research for investigating the tree carbon content variation between species [21], biomass components [22], growing sites [20], provenances [23], and forest origins [24]. Regardless of its impreciseness, the use of the 50% generic carbon multiplication factor seems to be endless, as often the very required specific carbon content value is still not available in the literature. Thus, fostering the investigation on carbon content measurement and variability is essential to promote global forest carbon stock assessments’ accuracy.

Food Agricultural and Organization of the United Nations (FAO) reported that, from 2010 to 2015, there was an increasing and a decreasing trend of global plantations and natural forest areas, with the rate of 3.3 million ha/year and 6.5 million ha/year, respectively [2,25]. Furthermore, researchers have found various palpable characteristic differences between these two origins, such as in terms of water consumption [26], carbon sequestration rate [27], soil organic carbon content [28], and species composition [29]. However, only a limited number of the established tree-level biomass functions used the data obtained from both plantations and natural stands to consider the origin’s effect in constructing the models. Fu et al. [30] used the aboveground biomass data of *Pinus massoniana* from planted and natural forests in southern China and concluded that involving the origin-effect with both dummy variables and mixed-effect models brought significantly better performance on model estimation compared to the population average model. Schuller et al. [31] harvested small diameter natural- and planted-origin trees (0.5–15 cm) of *Pinus taeda* in southern Arkansas to analyze the origin’s effect on biomass allometric relationships and reported that the partitioning proportions of branches, foliage, and root biomass were found to be greater in planted *Pinus taeda* compared to those of naturally regenerated samples, whereas this was vice versa for stems, aboveground, and total biomass. Widagdo et al. [24] utilized the above- and below-ground biomass dataset to establish *Larix gmelinii* origin-based individual tree biomass models in northeast China; and found that the origin-based models significantly improved the estimation of total biomass and all biomass components compared to the basic origin-free models. Further studies are necessitated to scrutinize the importance of involving the stands origin’s effect on biomass models and understanding the two habitats’ individual tree biomass characteristic’s variations.

Korean pine (*Pinus koraiensis*) is one of the most valuable evergreen needle-leaved tree species in northeast China, having a high quality of relatively decay-resistant timber [32] along with edible nutritional-rich [33] and unique-aroma perceived nuts [34]. In China, the natural and planted Korean pine respectively comprise an approximate total area of ~60 and ~300 thousand ha [35], designating the country as the world’s leading pine nut producer, with an estimated export value exceeding 250 million USD/year [36]. Furthermore, more than 98% of those natural and plantation Korean pine forest areas are located in northeast China, particularly in Heilongjiang province (69% cf. 49%), the most extensive among other regions. However, none of the previously established equations for *Pinus koraiensis* considered the forest origin’s effect, in which the majority of them only utilized either plantations or naturally regenerated sample trees. Hence, this study aimed to: (1) use the dummy variable with weighted NSUR model to involve the forest origin’s effect in developing additive biomass equations for the foliage, branch, stem, root, and total biomass of *Pinus koraiensis*; (2) utilize the jackknifing technique to validate the model fitting performance; (3) investigate the forest origin’s effect on biomass allocations, carbon content variability, and root-to-shoot (RS) ratio of *Pinus koraiensis*; and (4) compare our origin-free and -based biomass equations with the established models for Korean pine by Wang et al. [37] and Li et al. [38]

## 2. Materials and Methods

### 2.1. Data Collection

**Figure 1:**
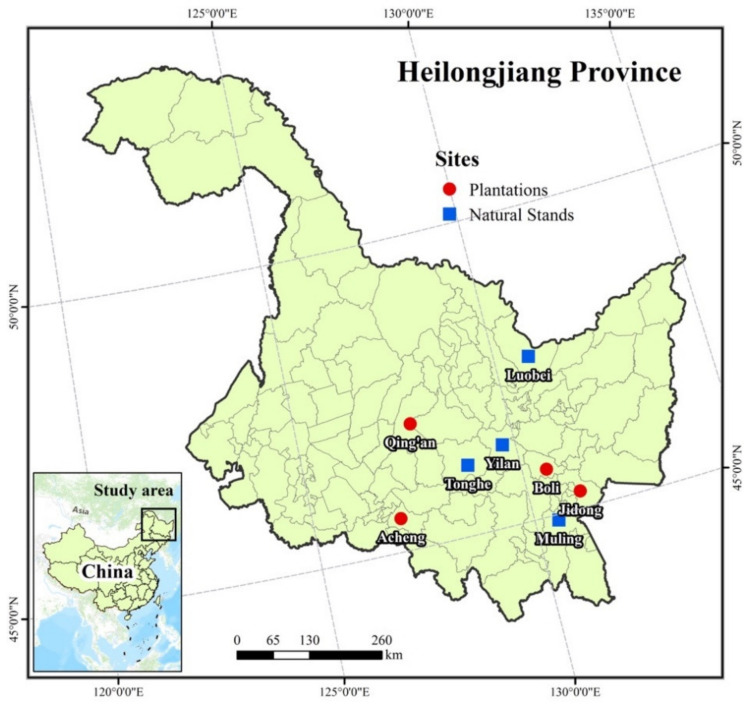
The location of the study area across several *Pinus koraiensis* plantations and natural stands in Heilongjiang Province, Northeast China.

The biomass and carbon content dataset used in the current research was obtained from both natural secondary forests (4 sites) and *Pinus koraiensis* plantations (4 sites) in the Xiaoxing’an and Changbai Mountains, Northeast China (Figure 1). All of the eight sites are located in Heilongjiang province (121–135° E and 43–53° N), the largest province in the northeastern area of China, where both natural and planted Korean pine was mostly situated. All sampling sites are owned and managed by The Forestry and Grassland Administration of Heilongjiang province, which hierarchically falls under the supervision of The State Forestry and Grassland Administration. The climate of the study areas is a continental monsoon climate (Köppen-Geiger climate classification *Dwb*), characterized by having warm-wet summers and dry-cold winters [39]. The mean annual rainfall ranges from 550 to 900 mm (with an average of ~700 mm). Based on the Chinese Taxonomic system, the soils are mostly dark brown forest soil (Haplumbrepts or Eutroboralfs). The mean annual temperature also ranges from −7 to 3 °C, with the mean temperature at the hottest (July) and coldest month (January) being 23 °C and −24 °C, respectively.

All planted trees were destructively sampled from the first rotation of *Pinus koraiensis* monoculture plantations, with the planting density of 2500 or 3300 trees/ha. The young stands were normally pruned once before reaching 20 years of age, and the middle-age stands were generally thinned one to three times with 5–7 years interval between each thinning practice (thinning intensity 20–25%). Meanwhile, all naturally regenerated sample trees were obtained from natural secondary forest sites, which are widespread across northeast China. This type of forest mostly has a great potential to become a primary forest, consisting of several layers of different ages, and Korean pine mostly occupies the canopy’s upper position.

Sixty-three Korean pine trees with DBH and H of 5.4–34.4 cm and 5.4–19.7 m, respectively, were destructively sampled. The biomass samples consisted of the aboveground (i.e., foliage, branch, and stem) and belowground (i.e., root) components from 33 naturally regenerated and 30 planted trees. Meanwhile, 45 trees were used for carbon content determination, in which 30 trees were obtained from plantations, and the balances were from natural stands. The field measurements were conducted in July and August when foliage biomass reaches its maximum. Some basic prerequisites were determined for selecting the sample trees, such as healthy, undamaged trees, those with an un-forked single trunk, and no border trees. Several parameters were measured in the field measurements: (1) DBH and H of each sampled trees; and (2) the green weight (kg) of foliage, branches, stems, and roots. Samples were then taken to the laboratory for oven drying and carbon content determination. The in-depth explanations regarding the field measurements and laboratory analysis have been entirely discussed in Dong et al. [14,22,40]. The descriptive statistics of the measured dry weight biomass and the independent variables (i.e., DBH and H) are presented in Table 1.

### 2.2. Statistical Analysis

#### 2.2.1. Base Model

**Figure 2:**
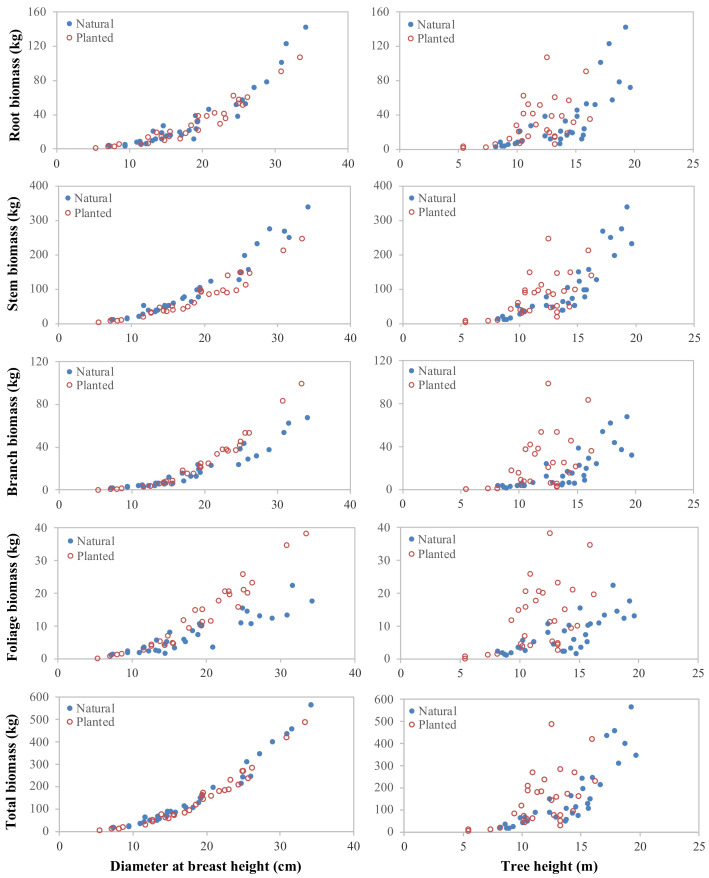
Relationship between the independent and dependent variables.

A scatterplot between the biomass data and available independent variables (i.e., *D* and *H*) was used to identify trends in the relationship (Figure 2). The base model was constructed using the common allometric power function with an additive error structure to examine the relationship between the predictors and biomass components. The equation can be written as follows:

(1) $$Y_{i}=e^{\beta_{i0}}X_{1}^{\beta_{i1}}X_{j}^{\beta_{i2}}+\varepsilon_{i}$$

where *Y_i_* denotes the *i*th biomass tissues in kg (*i* = 1st–4th respectively represent the root, stem, branch, and foliage); *X_i_* are the independent variables (*j* = 1 for *D* and *j* = 2 for *H*); *β_ij_* are the parameters of the models; *ε_i_* represents the error term. Considering one and two predictors to consist of *D* and a combination of *D* and *H*, respectively, has become a common practice in developing individual tree biomass models [16,41,42]. Thus, in this study, both uni- and multi-variate additive models were developed, providing more alternatives for forestry practitioners and researchers.

#### 2.2.2. Population Average (Generalized) Model

The population average (PA) models were fitted to all data without considering the effects of tree origin (i.e., plantation and natural forest). Cross-equation with one parameter restriction was used to account for the compatibility property within each five-equation system as it has been suggested by Parresol [15]. This structure ensures the additivity between the estimation of the component biomass, making it possible to sum a certain component’s estimation depending on the necessity. The additive systems of the PA models with one (*PA*-1) and two (*PA*-2) predictors can be expressed as:

(2) $$PA-1\left\{\begin{array}{c} Y_{r}=e^{\beta_{r0}}D^{\beta_{r1}}+\varepsilon_{r}\\ Y_{s}=e^{\beta_{s0}}D^{\beta_{s1}}+\varepsilon_{s}\;\;\\ Y_{b}=e^{\beta_{b0}}D^{\beta_{b1}}\varepsilon_{b}\\ Y_{f}=e^{\beta_{f0}}D^{\beta_{f1}}+\varepsilon_{f}\\ Y_{t}=Y_{r}+Y_{s}+Y_{b}+Y_{f}+\varepsilon_{t}\;\; \end{array}\right.$$

(3) $$PA-2\left\{\begin{array}{c} Y_{r}=e^{\beta_{r0}}D^{\beta_{r1}}H^{\beta_{r2}}+\varepsilon_{r}\\ Y_{s}=e^{\beta_{s0}}D^{\beta_{s1}}H^{\beta_{s2}}+\varepsilon_{s}\;\;\\ Y_{b}=e^{\beta_{b0}}D^{\beta_{b1}}H^{\beta_{b2}}+\varepsilon_{b}\\ Y_{f}=e^{\beta_{f0}}D^{\beta_{f1}}H^{\beta_{f2}}+\varepsilon_{f}\\ Y_{t}=Y_{r}+Y_{s}+Y_{b}+Y_{f}+\varepsilon_{t}\;\; \end{array}\right.$$

Where *Y_r_, Y_s_, Y_b_,* and *Y_f_* respectively denote the root, stem, branch, foliage, and total biomass. Other abbreviations are the same with Equation (1).

#### 2.2.3. Origin-Based Dummy Variable Model

Forest origins can be considered as categorical effects in constructing biomass models [9,24,30,43]. Hence, we used a dummy variable (DV) model to account for the differences of the specific traits between the naturally regenerated and planted single-tree biomass model. As in PA models, the NSUR with one parameter restriction was also used to guarantee the presence of the additivity property between each component’s biomass equation. The additive systems of the DV models with one (*DV*-1) and two (*DV*-2) predictors can be expressed as:

(4) $$DV-1\left(\begin{array}{c} Y_{r}=e^{\left(\beta_{r0i}*Z_{0i}+\beta_{r0i}*Z_{0i}\right)}D^{\left(\beta_{r1i}*Z_{1i}+\beta_{r1i}*Z_{1i}\right)}+\varepsilon_{r}\\ Y_{s}=e^{\left(\beta_{s0i}*Z_{0i}+\beta_{s0i}*Z_{0i}\right)}D^{\left(\beta_{s1i}*Z_{1i}+\beta_{s1i}*Z_{1i}\right)}+\varepsilon_{r}\\ Y_{b}=e^{\left(\beta_{b0i}*Z_{0i}+\beta_{b0i}*Z_{0i}\right)}D^{\left(\beta_{b1i}*Z_{1i}+\beta_{b1i}*Z_{z1i}\right)}+\varepsilon_{r}\\ Y_{f}=e^{\left(\beta_{f0i}*Z_{0i}+\beta_{f0i}*Z_{0i}\right)}D^{\left(\beta_{f1i}*Z_{1i}+\beta_{f1i}*Z_{1i}\right)}+\varepsilon_{r}\\ Y_{t}=Y_{r}+Y_{s}+Y_{b}+Y_{f}+\varepsilon_{t} \end{array}\right)$$

(5) $$DV-2\left(\begin{array}{c} Y_{r}=e^{\left(\beta_{r0i}*Z_{0i}+\beta_{r0i}*Z_{0i}\right)}D^{\left(\beta_{r1i}*Z_{1i}+\beta_{r1i}*Z_{1i}\right)}H^{\left(\beta_{r2i}*Z_{2i}+\beta_{r2i}*Z_{2i}\right)}+\varepsilon_{r}\\ Y_{s}=e^{\left(\beta_{s0i}*Z_{0i}+\beta_{s0i}*Z_{0i}\right)}D^{\left(\beta_{s1i}*Z_{1i}+\beta_{s1i}*Z_{1i}\right)}H^{\left(\beta_{s2i}*Z_{2i}+\beta_{s2i}*Z_{2i}\right)}+\varepsilon_{r}\\ Y_{b}=e^{\left(\beta_{b0i}*Z_{0i}+\beta_{b0i}*Z_{0i}\right)}D^{\left(\beta_{b1i}*Z_{1i}+\beta_{b1i}*Z_{1i}\right)}H^{\left(\beta_{b2i}*Z_{2i}+\beta_{b2i}*Z_{2i}\right)}+\varepsilon_{r}\\ Y_{f}=e^{\left(\beta_{f0i}*Z_{0i}+\beta_{f0i}*Z_{0i}\right)}D^{\left(\beta_{f1i}*Z_{1i}+\beta_{f1i}*Z_{1i}\right)}H^{\left(\beta_{f2i}*Z_{2i}+\beta_{f2i}*Z_{2i}\right)}+\varepsilon_{r}\\ Y_{t}=Y_{r}+Y_{s}+Y_{b}+Y_{f}+\varepsilon_{t} \end{array}\right)$$

where *β_rji_*, *β_sji_*, *β_bji_*, and *β_fji_* are respectively the origin-specific coefficients for roots, stems, branches, and foliage biomass (*j* = *0*, *1*, and *2*; *i* = *a* and *b*); *Z_ji_* represents the dummy variable; and other abbreviations are the same with in Equations (1)–(3). The dummy variable *Z_ji_* was set as follows: *Z_ja_* = 1 and *Z_jb_* = 0 for the data acquired from Korean pine plantation, while *Z_ja_* = 0 and *Z_jb_* = 1 for those obtained from natural forest.

#### 2.2.4. Heteroscedasticity Neutralization

Heteroscedasticity is a common problem in tree biomass and volume modeling, which creates a certain pattern of inconsistency in the model’s residuals. To date, two methods (i.e., logarithmic transformation and weighted regression) have been widely applied as countermeasures to reduce the heteroscedasticity issue [16]. In the present study, the weighted least squares regression was used to homogenize the error variance, since it is more reliable to be applied for the nonlinear regression. For each biomass equation, a unique weighting function was determined using the power function to independently fit the predictors (*D_i_*) and the error variance (*ε_i_*) of the ith individuals. A more detailed theoretical explanation can be found in Harvey [44], while the practical application using the SAS/ETS PROC MODEL procedure [45] in Dong et al. [14] and Balboa-Murias et al. [46]

#### 2.2.5. Model Validation

The two additive model systems (PA and DV) were fitted using the entire dataset (sample size N). In the present study, there were no independent data available for model validation; thus, the jackknifing technique (leave-one-out) was utilized. All exclude-one observations (sample size N−1) were systematically utilized in building the validation models, and the held-out observation was used to calculate the predictions. Two statistics were employed for model fitting assessment: (1) the coefficient of determination (*R*^2^); and (2) root mean square error (RMSE), while three jackknifing statistics were utilized for evaluating the model performance: (1) mean error (ME); (2) mean absolute error (MAE); and (3) mean absolute percentage error (MAPE). The equations can be written as follows:

(6) $$R^{2}=1-\frac{\sum_{i=1}^{n}{\left(Y_{ik}-{\hat{Y}}_{ik}\right)}^{2}}{\sum_{i=1}^{n}{\left(Y_{ik}-{\bar{Y}}_{k}\right)}^{2}}$$

(7) $$\mathrm{RMSE}=\sqrt{\frac{\sum_{i=1}^{n}{\left(Y_{ik}-{\hat{Y}}_{ik}\right)}^{2}}{n}}$$

(8) $$\mathrm{ME}=\frac{\sum_{i=1}^{n}\left(Y_{ik}-{\hat{Y}}_{ik,-i}\right)}{n}$$

(9) $$\mathrm{MAE}=\frac{\sum_{i=1}^{n}\left|Y_{ik}-{\hat{Y}}_{ik,-i}\right|}{n}$$

(10) $$\mathrm{MAPE}=\frac{\sum_{i=1}^{n}\left|(Y_{ik}-{\hat{Y}}_{ik,-i})/Y_{ik}\right|}{n}\times 100$$

where *Y_ik_* is the observed biomass for the *i*th individual and the *k*th category (*k* = 1, 2, 3, 4, and 5, are root, stem, branch, foliage, and total, respectively); *Ŷ_ik_* is the predicted biomass for the *i*th individual and the *k*th category (fitted using N sample size); *Ŷ* is the average value of the observed biomass; and *Ŷ_ik,−i_* is the estimated biomass for the *i*th individual and the *k*th category (fitted using N−1 sample size). Furthermore, to analyze whether or not the effect of considering growing origin in the dummy variable (DV) models brought a significant improvement compared to the population average (PA) models, both *F*-test and Lakkis-Jones test [47,48] were applied in this study. The statistics were calculated as follow:

(11) $$F=\;\left(\frac{SSE_{PA}-SSE_{DV}}{d.f._{PA}-d.f._{DV}}\right)\times \frac{d.f._{DV}}{SSE_{DV}}$$

(12) L=2lnSSEPASSEDVN2

where *SSE_PA_* and *SSE_DV_* denote the population average and dummy variable models (without and with growing origin variables) sum of squares errors, respectively; *d.f._PA_* and *d.f._DV_* represent the degrees of freedom of the PA and DV models, respectively; and *N* is the total samples.

#### 2.2.6. Variation of Carbon Content

The factorial analysis of variance (ANOVA) test was used to assess the effect of tree size (*D*), tree height (*H*), growing origins (i.e., plantation and natural forest), and biomass components (i.e., foliage, branch, stem, and root) on carbon content value. The analysis was conducted using the SAS PROC GLM procedure [45], as it has been commonly performed in previous researches [20,49,50].

## 3. Results

### 3.1. Biomass Partitioning between the Planted and Naturally Regenerated Korean Pine

**Figure 3:**
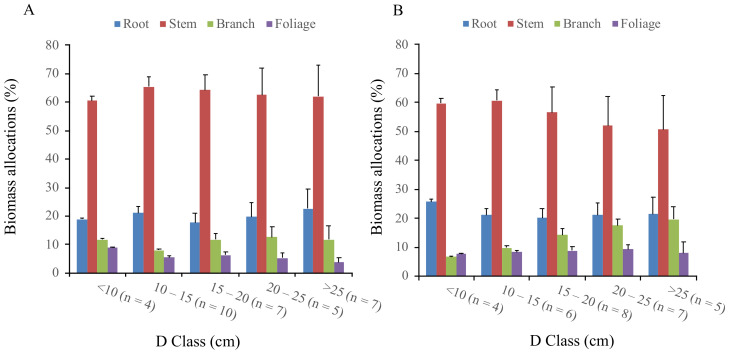
Biomass allocation of the four primary components across five range of diameter classes for: (**A**) The naturally regenerated; and (**B**) the planted *Pinus koraiensis.* The bars are standard errors and n is the number of sample trees per D class.

Figure 3 presents the biomass relative percentage of each part (i.e., foliage, branch, stem, and root) across five diameter (D) classes (i.e., <10, 10–15, 15–20, 20–25, >25) as Ruiz–Peinado et al. [51] noted that the distribution of biomass into different parts was highly related to D. The belowground biomass (root) relative to the proportion of the total biomass was consistent in planted Korean pine, around 22% in five diameter classes. For the naturally regenerated Korean pine, the root biomass proportion did not indicate any regular pattern across five diameter classes (Figure 3). The stem biomass relative proportion to total biomass was found to decrease gradually from the 2nd to the 5th D classes in both origins. However, the decline was almost three times higher in Korean pine plantations (~10%) than in natural stands (~3.5%). The branch biomass proportion of the planted Korean pine increased gradually from the 1st (6.8%) to the 5th (19.6%) D classes. For the natural Korean pine, apart from the 2nd D class (8.02%), branch biomass presents a consistent relative percentage (~12%). The same pattern occurred in the foliage component of the planted sample trees, in which the foliage biomass percentage was found to be constant at around 8.5% across all D classes (Figure 3). Meanwhile, for natural Korean pine, a decreasing trend in foliage biomass proportion was shown from the 1st (8.8%) to the 5th (3.8%) D classes.

### 3.2. RS Ratio and Carbon Content Variations of the Two Origins

**Figure 4:**
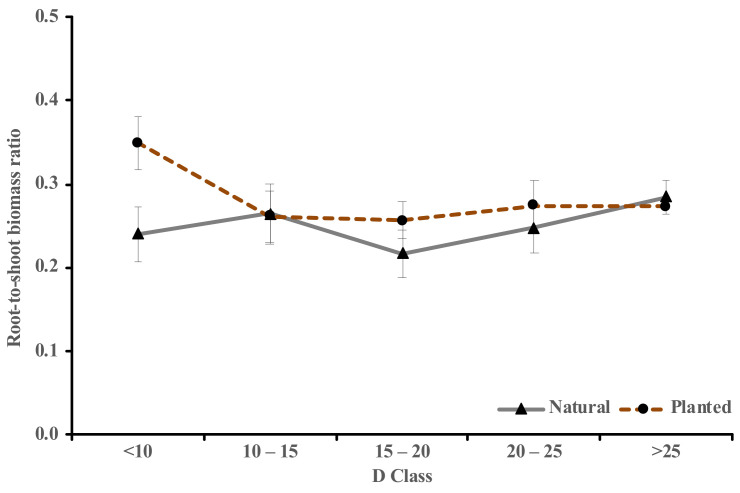
Comparison of the root-to-shoot (RS) ratio between the planted and natural *Pinus koraiensis* across five ranges of diameter classes. The bars are standard errors. The number of the sample trees per D class is similar with Figure 3.

The pattern of the root-to-shoot (RS) ratio was also visualized across five diameter classes based on the tree growing origins (Figure 4). The 1st D class of the planted sample trees gave the highest RS ratio (0.35), while the 3rd D class of the naturally regenerated Korean pine accounts for the smallest RS ratio (0.22). For the planted sample trees, apart from the 1st D class, the RS ratio was showing a regular pattern from the 2nd to the 5th D classes (0.27 ± 0.01). Meanwhile, the RS ratio pattern of the naturally regenerated sample trees was found to be irregular across the five D classes (0.24, 0.26, 0.22, 0.25, and 0.28). On average (±SD), the RS ratio of the planted Korean pine (0.28 ± 0.04) was found to be slightly higher than the naturally regenerated sample trees (0.25 ± 0.03).

**Figure 5:**
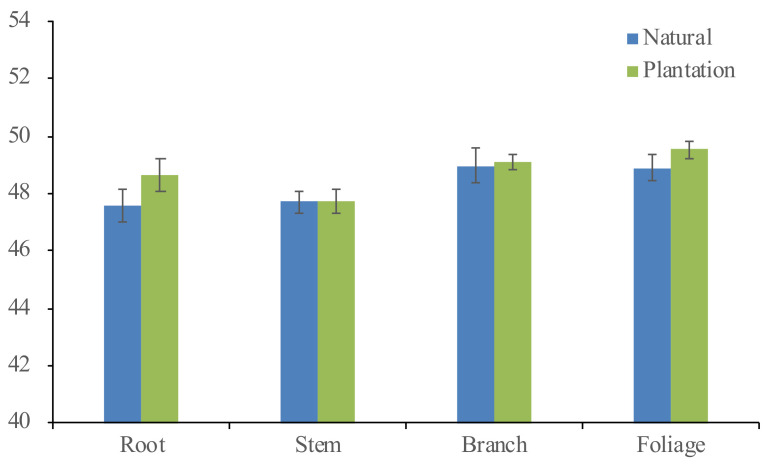
The carbon content of *Pinus koraiensis* for the root, stem, branch, and foliage sampled from both natural and plantation forests. Means are equal to the height of the bars, while the vertical lines represent the standard error.

The carbon content of foliage, branch, stem, and root biomass tissues from both origins (plantations and natural stands) were also measured in this study (Figure 5). The effect of tree DBH (F value = 14.20 and *p* = 0.0002), tree total height (F value = 5.19 and *p* = 0.0240), and biomass tissues (F value = 5.71 and *p* = 0.0009) on carbon content were found to be significantly different. Meanwhile, the effect of growing origins (F value = 0.40 and *p* = 0.5275) and all interactions between variables (i.e., tree DBH, tree total height, origins, and biomass tissues) were found to be not statistically significant. The overall carbon content can be ranked: foliage (49.32%) > branch (49.04%) > root (48.28%) > stem (47.73%). The planted Korean pine had a slightly higher average carbon content than those of naturally regenerated sample trees (48.75% cf. 48.29%), as well as across all four primary tree tissues (Figure 5). However, the differences of all biomass tissues between the two origins were not statistically significant.

### 3.3. The PA and DV Additive Biomass Model Systems

The data of 63 destructively sampled *Pinus koraiensis* from Northeast China were used to develop one- and two-variable biomass models. Using the PROC MODEL procedure in SAS Software (SAS Institute Inc., Cary, NC, USA, 2011), the population average (PA) and dummy variable (DV) additive biomass model systems were simultaneously fitted by applying one constraint in the NSUR model structure to ensure the presence of the additivity property (Equations (2)–(5)). The fitting results of PA and DV model systems for the four primary components (i.e., stem, foliage, branch, and root) and total biomass are presented in Table 2 and Table 3, respectively.

The parameters of D were positive in all equations (Table 2 and Table 3). Meanwhile, the parameters of H varied depending on the biomass tissues. The parameters of H in all of the stem and foliage equations (i.e., PA-2 and DV-2) were always found to be positive and negative, respectively. The stem tissue’s positive relationship with both parameters D and H indicated that, for an equivalent value of D, the stem biomass rose with rising H. The positive D and negative H in the foliage tissue implicated that, for an equivalent value of D, foliage biomass decreased with the rising H. For the branch tissue, the results varied between origins (Table 3). In plantation sites, the parameter of H was found to be positive, indicating the branch biomass rose with rising H for an equivalent value of D, while this was vice versa in natural forests. Meanwhile, different results were obtained between the PA and DV models for the root biomass equations, in which the parameter H was found to be positive in the PA model and negative in both the DV models (plantation and natural sites).

**Figure 6:**
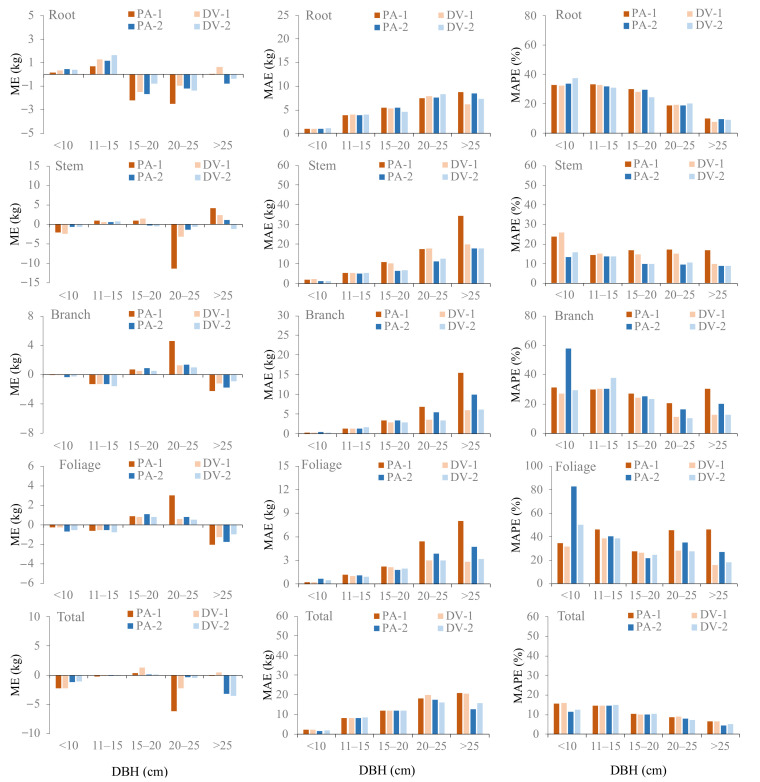
Comparison of the leave-one-out validation results between the additive system of population average models (PA-1 and PA-2) and dummy variable models (DV-1 and DV-2) across five ranges of diameter classes. ME: mean error; MAE: mean absolute error; MAPE: mean absolute percentage error.

Table 4 showed that the two additive biomass model systems (i.e., PA and DV) developed in this study satisfactorily fitted the data. The foliage tissue had the lowest model fitting (*R*^2^ = 0.699–0.930), while total biomass models delivered the best results of model fitting (*R*^2^ = 0.985–0.989). The addition of H into two-variable biomass equations gave an apparent improvement in the models’ performances compared to the one-predictor models, successfully increasing the models’ *R*^2^ and decreasing the ME, MAPE, MAE, and RMSE (Table 4). More detailed model validations are presented in Figure 6, where the MAPE, ME, and MAE were visualized throughout five ranges of D classes. The figure discovered that DV mostly yielded smaller errors than PA, indicating that better model performance was provided by DV models. PA-1 often appeared as the worst model, and DV-2 as the best model across all diameter classes and biomass tissues (Figure 6). Overall, the model performances can be ranked according to the combination of the model predictors as follows: DV-2 > PA-2 and DV-1 > PA-1.

The significant differences between the origin-free and -based biomass models were tested using both Lakkis Jones (*L*-test) and nonlinear extra sum of squares tests (*F*-test; Table 4). Apart from DV-2 in stem biomass equations, the *F*-test showed significantly different results (*p* < 0.0001) between the PA and DV models across all of the four primary tree tissues (*p* < 0.005 for DV-1 in root biomass). Meanwhile, *F*-values of both DV-1 and DV-2 in total biomass equations were found to be insignificant. However, the *L*-test showed significant differences between the PA and DV additive model systems for foliage, branch, stem, and root biomass (*p* < 0.0001), including DV-1 (*p* < 0.001) and DV-2 (*p* < 0.05) in total biomass equations. These results indicated that tree growing origin is an essential element to be considered in developing tree-level biomass models for *Pinus koraiensis*. However, our newly developed PA models are still able to be used; even the performance was not as accurate as the DV models.

### 3.4. Comparison with the Established Pinus Koraiensis Biomass Models

**Figure 7:**
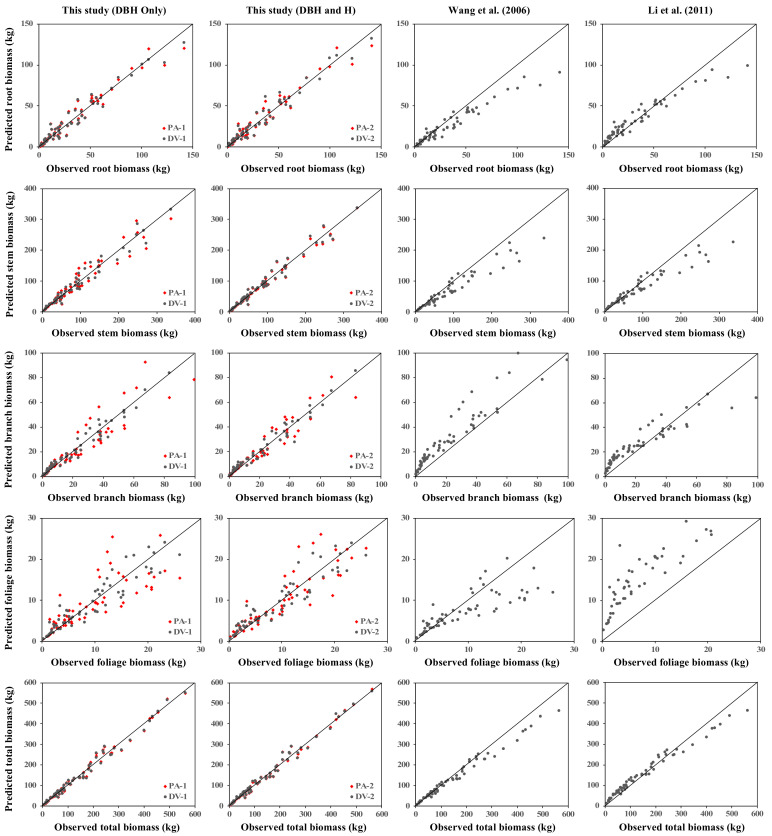
Comparison between the models developed in this study and the previously published biomass models for Korean pine.

All additive equation systems (i.e., PA-1, PA-2, DV-1, and DV-2) were compared against the established equations for predicting single-tree biomass of Korean pine by Wang et al. [37] and Li et al. [38]. The prediction results of each model were plotted against the observed biomass value in Figure 7. Both one- and two-predictors equations of PA and DV models gave apparent better performances compared to the two previously published equations. DV-2 constantly presented the lowest RMSE for the root (6.44 kg), stem (11.68 kg), branch (3.48 kg), foliage (2.25 kg), and total biomass (15.58 kg). Meanwhile, the highest RMSE values were yielded by the equations of Wang et al. [37] for root (12.73 kg), branch (11.32 kg), and total biomass (29.58 kg), and similarly with the equations of Li et al. [38] for stem (31.56 kg) and foliage biomass (11.93 kg). Furthermore, both Wang et al. [37] and Li et al. [38] equations had relatively high relative errors (RE), which specifically generated remarkable overestimations for branch (98.94%) and foliage biomass (185.89%), respectively (Figure 7).

## 4. Discussion

Biomass partitioning has been acknowledged as an important element in plant physiological ecology as it reflects the plant’s long-term responses to various environmental conditions [52,53] and thus will potentially have a more critical role in the future. We observed the variations in biomass allocation between the planted and naturally regenerated *Pinus koraiensis* across five ranges of D classes. The branch biomass proportions in Korean pine plantation yielded a ~10% increase from the 2nd to the 5th D class (Figure 3). The reason is likely related to one of the major purposes of Korean pine plantation establishment in Northeast China, pine nut production [33,54]. Hence, the branch became thicker, larger, and stronger to support the high number of produced cones. The average partitioning proportions of stems (62.9 ± 1.9%) in the natural environment were ~7.0% higher than those found in plantations (56.0 ± 4.4%) due to having ~2 m more in average tree total height across the same ranges of D (Table 1). These results might indicate that the natural Korean pine trees obtained lower light intensities compared to those grown in plantations [55,56,57]. As the “functional equilibrium” theory explained that if the limiting factor is located above-ground, plants will relatively allocate more biomass in shoots (i.e., foliage, branch, and stem), and vice versa if the growth limiting factor is located below-ground [58,59].

The root-to-shoot (RS) biomass ratio was quantified for *Pinus koraiensis* since it has been commonly known as a simple option to allometric equations for predicting below-ground biomass [52,60,61]. Both mean (0.27 ± 0.03) and ranges (0.22–0.35) of the obtained RS ratio are located within the RS ratio ranges (0.18–0.35) of other pine and coniferous tree species [61,62]. Planted Korean pine had a slightly insignificantly higher RS ratio than those of naturally regenerated sample trees (0.28 cf. 0.25), different from previous research by Luo et al. [63], which reported that natural forests had significantly greater RS ratio compared to plantations. However, their forest-origin comparison dataset consisted of several mixed broadleaf-coniferous tree species across China’s forests, ignoring the species-specific variation. Pooling all species to analyze the RS ratio difference between origins might yield uncertainties, as the opposite result was obtained by Mokany et al. [52]. They used a pooled dataset from global forests and concluded that no significant differences were identified between the RS ratio of the plantations and natural forests. Thus, the species-specific RS ratio is more reliable, since each tree species has different growth strategies, architecture, and vibrant interaction with various biophysical environments [64,65].

Apart from the tree growing origins (i.e., plantation and natural forest), the carbon content of *Pinus koraiensis* was found to be affected significantly by DBH, H, and biomass tissues (i.e., stem, root branch, and foliage). The carbon content of *Pinus koraiensis* was the highest in foliage, slightly lower in branches, the lowest in stems, and slightly higher in roots. A statistically significant difference in carbon content between the four primary tree tissues has also been reported in the literature for other tree species in Northeast China [20,22]. Furthermore, Rodríguez-Soalleiro et al. [21] revealed that tree species and biomass tissues are considered the biggest sources in tree carbon content variation, explaining about 69% of the total variance. In addition, carbon content of planted *Pinus koraiensis* was slightly insignificantly higher compared to the naturally regenerated trees, both averagely (48.75% cf. 48.29%) and separately across the foliage (49.54% cf. 48.90%), branches (49.08% cf. 48.98%), roots (48.64% cf. 47.57%), and stems (47.75% cf. 47.69%). Similar results were obtained by Elias and Potvin [66], which analyzed the carbon content difference between the two origins using ten tropical tree species from Panama. It turns out that seven species were insignificantly different, in which three of them (*Anacardium excelsum*, *Enterolobium cyclocarpum*, and *Tabebuia rosea*) had higher carbon content in plantations than natural forests. Overall, the average carbon content of Korean pine (48.60%) obtained in this study had a 1.40% lower value than the commonly used 50% carbon content. Hence, using the 50% generic carbon content might yield an approximately 1.0–4.5% overestimated prediction for tree-level Korean pine carbon stock quantification. A more detailed analysis regarding the bias yielded by the widely used 50% carbon constant value can be seen in Zhang et al. [50], Dong et al. [40], and Widagdo et al. [20].

As one of the most superior predictors for predicting biomass, diameter at breast height (D) has been widely applied in a large number of tree-level species’ allometric equations across regions and forest types [7,31]. However, researchers still put a strong effort into increasing the biomass equation’s accuracy by adding more supporting independent predictors, such as tree total height (H), crown length, crown radius, wood density, and tree/stand age [6,19,41]. Among those, H has been reported as the most promising additional predictor to D, introducing a remarkable improvement on the model’s predictive ability. Our results are consistent with the literature, in which the addition of H mostly increases the models’ fitting performance across the five model categories (i.e., total, stem, root, branch, and foliage; Table 3). In addition, the relationship between crown biomass and H was found to be negative, as has been repeatedly reported in previous studies [16,24]. It is indeed in full compliance with the natural law of the forest: for trees having the same D, shorter trees generally tend to have a denser and larger crown compared to those of higher trees, yielding more biomass allocated in the crown [67,68].

The effects of tree-growing origins (plantations and natural forests) were also considered by developing the dummy variable (DV) models and comparing the biomass prediction results with the origin-free population average (PA) models. Significantly different results were obtained between the DV and PA models (Table 3), suggesting that the origin’s effect needs to be properly considered to establish biomass equations for *Pinus koraiensis*. Compared to the PA models, the DV models successfully reduced the estimation bias by 35.80%, 41.65%, 12.57%, and 6.35%, and for the foliage, branch, stem, and root biomass, respectively (calculated using RMSE, Equation (7)). These findings are in line with previous research by Zeng [9], Schurel et al. [31], and Widagdo et al. [24], which confirmed the importance of considering the origin’s effect on biomass models of Chinese fir (*Cunninghamia lanceolata*), loblolly pine (*Pinus taeda*), and larch (*Larix gmelinii*) in southern China, Arkansas, and northeast China, respectively. Better performance of origin-based biomass models might also be related to a major shift in productivity allocation (i.e., stand density or site productivity) between the planted and natural forest, leading to differences in their tree features, such as wood density [60].

In this study, all newly developed models (PA-1, PA-2, DV-1, and DV-2) were entirely compared against the previously established biomass equations for *Pinus koraiensis* (Figure 7), developed by Wang et al. [37] and Li et al. [38]. However, both of these researchers only used the dataset from the Korean pine monoculture plantations, neglecting the forest origin’s effect in constructing the models. Wang et al. [37] used 10 destructively sampled trees from northeast China, and Li et al. [38] felled 35 trees from central South Korea to establish several belowground and aboveground biomass tissue equations. The additivity property between several biomass tissues was also not considered in their developed equations, in which it has been properly considered in our both PA and DV additive model systems. Thus, remarkable errors were produced when their biomass equations fitted to our specific tissues and total biomass dataset, which consist of destructively sampled trees obtained from both plantations and natural stands. Overall, our uni- and multi-variable model systems provided high accuracy for predicting tree-level biomass of both planted and natural *Pinus koraiensis* compared to several previously established biomass equations for *Pinus koraiensis*.

## 5. Conclusions

Uni- and multi-variable biomass models were developed for the two additive systems of population average (PA) and dummy variable (DV) models, using destructively sampled biomass datasets of natural and planted *Pinus koraiensis* from northeast China. Forest origins were found to significantly affect the biomass equations, specifically for the four primary tree tissues. Incorporating the origins effect in DV models and introducing tree total height (H) as an additional predictor to DBH effectively improved the biomass equations’ accuracy. Besides being outperformed by the DV models, PA models presented relatively good performances and can be alternatively used when both natural and planted Korean pine data are an inseparable set. The property of additivity was also presented in our biomass model systems using nonlinear seemingly unrelated regression (NSUR) with specific weighted functions for each equation to solve the heteroscedasticity problem. All models have been validated using the Jackknifing technique. Overall, the performance of our newly developed PA and DV models can be ranked according to their respective combination of predictors: DV-2 > PA-2 and DV-1 > PA-1.

Biomass allocations, carbon content variability, and root-to-shoot (RS) ratio of the planted and natural Korean pine were also analyzed in this study. The differences in biomass distribution patterns between the two origins were apparent, and plantations had a slightly higher average value than natural forests in terms of RS ratio (0.28 cf. 0.25) and carbon content (48.75% cf. 48.29%). Distinct traits of biomass partitioning, carbon content, and RS ratio were detected between plantations and natural forests. Hence, forest origin is a crucial element to be considered for improving the accuracy of tree-level biomass equations and carbon stock estimations. Our newly established biomass model systems can be used for estimating the biomass and carbon of *Pinus koraiensis*, specifically for the National Forest Inventory of China. However, these equations will only deliver their best performance for usage within the appropriate data coverage (i.e., DBH, H, and regions); otherwise, a large bias might be produced.

## Figures and Tables

**Table 1:** Summary statistics of the tree samples.

Variables	Naturally Regenerated (N = 33)			Planted (N = 30)			Total (N = 63)		
	Mean	Range	S.D.	Mean	Range	S.D.	Mean	Range	S.D.
Tree DBH (cm)	18.2	7.3–34.4	7.3	18.4	5.4–33.4	6.9	18.3	5.4–34.4	7.0
Total height (m)	13.7	8.2–19.7	3.3	11.5	5.4–16.2	2.7	12.7	5.4–19.7	3.2
Root biomass (kg)	32.7	2.3–141.4	35.0	31.3	1.6–107.0	26.0	32.0	1.6–141.4	30.8
Stem biomass (kg)	97.3	8.3–336.9	87.9	79.1	3.4–246.5	60.0	88.6	3.4–336.9	75.8
Branch biomass (kg)	17.7	1.4–67.4	18.1	25.2	0.3–99.3	24.6	21.3	0.3–99.3	21.6
Foliage biomass (kg)	7.3	1.1–22.4	5.4	12.9	0.3–38.3	10.0	9.9	0.3–38.3	8.3
Total biomass (kg)	155.0	14.0–563.1	144.1	148.6	5.6–491.2	118.8	152.0	5.6–563.1	131.6

DBH: Diameter at breast height; S.D.: Standard deviation; N: Number of samples.

**Table 2:** Parameter estimates of the one- and two-variable population average (PA) additive models.

Equations	Components	Parameters	Estimates	S.E.
PA-1 (One-variable)	Root	$\beta_{r0}$	−4.10368	0.1765
		$\beta_{r1}$	2.52639	0.0548
	Stem	$\beta_{s0}$	−2.29469	0.2002
		$\beta_{s1}$	2.26941	0.0644
	Branch	$\beta_{b0}$	−5.40818	0.3448
		$\beta_{b1}$	2.79699	0.1066
	Foliage	$\beta_{f0}$	−3.73742	0.4135
		$\beta_{f1}$	2.01881	0.1325
PA-2 (Two-variables)	Root	$\beta_{r0}$	−4.47742	0.2138
		$\beta_{r1}$	2.51914	0.0720
		$\beta_{r2}$	0.14399	0.1051
	Stem	$\beta_{s0}$	−3.36539	0.2068
		$\beta_{s1}$	1.94692	0.0661
		$\beta_{s2}$	0.77835	0.0914
	Branch	$\beta_{b0}$	−4.14573	0.3243
		$\beta_{b1}$	3.13166	0.1041
		$\beta_{b2}$	−0.86857	0.1251
	Foliage	$\beta_{f0}$	−1.96307	0.3978
		$\beta_{f1}$	2.56451	0.1310
		$\beta_{f2}$	−1.31504	0.1849

S.E. is the standard error.

**Table 3:** Parameter estimates of the one- and two-variable dummy variable (DV) additive models.

Equations	Origins	Components		Parameters	Estimates	S.E.
DV-1 (One-variable)	Natural	Root		$\beta_{r0}$	−4.58785	0.3204
				$\beta_{r1}$	2.67952	0.0994
		Stem		$\beta_{s0}$	−2.25782	0.2153
				$\beta_{s1}$	2.28230	0.0652
		Branch		$\beta_{b0}$	−4.95561	0.3411
				$\beta_{b1}$	2.60134	0.1051
		Foliage		$\beta_{f0}$	−3.18675	0.4247
				$\beta_{f1}$	1.75082	0.1385
	Planted	Root		$\beta_{r0}$	−3.81245	0.3607
				$\beta_{r1}$	2.41863	0.1154
		Stem		$\beta_{s0}$	−2.23070	0.2973
				$\beta_{s1}$	2.20883	0.0918
		Branch		$\beta_{b0}$	−5.82831	0.3084
				$\beta_{b1}$	2.99239	0.0953
		Foliage		$\beta_{f0}$	−4.26419	0.3503
				$\beta_{f1}$	2.27914	0.1114
DV-2 (Two-variables)	Natural	Root		$\beta_{r0}$	−4.24899	0.5111
				$\beta_{r1}$	3.02592	0.2160
				$\beta_{r2}$	−0.51898	0.3727
		Stem		$\beta_{s0}$	−3.40236	0.3582
				$\beta_{s1}$	1.85305	0.1258
				$\beta_{s2}$	0.90319	0.2300
		Branch		$\beta_{b0}$	−3.63392	0.5838
				$\beta_{b1}$	3.07384	0.2351
				$\beta_{b2}$	−1.01454	0.4008
		Foliage		$\beta_{f0}$	−1.53751	0.8336
				$\beta_{f1}$	2.34938	0.4170
				$\beta_{f2}$	−1.27838	0.6761
	Planted	Root		$\beta_{r0}$	−3.64574	0.3971
				$\beta_{r1}$	2.47972	0.1166
				$\beta_{r2}$	−0.14226	0.2108
		Stem		$\beta_{s0}$	−3.33091	0.4193
				$\beta_{s1}$	2.05227	0.0976
				$\beta_{s2}$	0.62892	0.1616
		Branch		$\beta_{b0}$	−5.77974	0.4322
				$\beta_{b1}$	2.94660	0.1052
				$\beta_{b2}$	0.03947	0.1565
		Foliage		$\beta_{f0}$	−3.95709	0.6063
				$\beta_{f1}$	2.25431	0.1504
				$\beta_{f2}$	−0.08953	0.2446

S.E. is the standard error.

**Table 4:** Weighting functions, model validation statistics, and the Lakkis and Jones (*L*-test) and the non-linear extra sum of squares (*F*-test) results in an investigation of the differences between the PA and DV models.

Models	Weighting Functions	*R* ^2^	RMSE	ME	MAE	MAPE	*L*-Value	Pr > |*L*|	*F*-Value	Pr > |*F*|
**Root**										
PA-1	D^3.5046^	0.948	7.02	−0.82	5.41	25.76				
DV-1	D^2.2508^	0.956	6.51	−0.08	5.03	24.79	50.06	<0.0001	11.04	0.0015
PA-2	D^2.6015^	0.952	6.81	−0.45	5.40	25.38				
DV-2	D^3.0141^	0.958	6.44	−0.05	5.16	24.55	41.56	<0.0001	5.92	<0.0001
**Stem**										
PA-1	D^2.1503^	0.936	19.25	−1.03	13.72	17.28				
DV-1	D^1.1556^	0.963	14.75	0.14	11.01	15.50	165.33	<0.0001	44.65	<0.0001
PA-2	D^1.4699^	0.976	11.89	−0.01	8.36	11.08				
DV-2	D^1.4078^	0.977	11.68	−0.32	8.76	11.57	18.07	<0.0001	2.47	0.1209
**Branch**										
PA-1	D^1.7870^	0.884	7.37	0.28	5.22	27.69				
DV-1	D^1.8359^	0.972	3.65	−0.21	2.86	21.90	428.88	<0.0001	192.59	<0.0001
PA-2	D^1.7992^	0.943	5.18	−0.23	4.03	28.09				
DV-2	D^1.5032^	0.975	3.48	−0.31	2.88	23.65	245.76	<0.0001	50.50	<0.0001
**Foliage**										
PA-1	D^1.5015^	0.699	4.58	0.20	3.28	39.59				
DV-1	D^1.9542^	0.927	2.72	−0.08	1.87	28.65	426.82	<0.0001	190.86	<0.0001
PA-2	D^1.6854^	0.848	3.26	−0.09	2.35	37.42				
DV-2	D^1.6308^	0.930	2.25	−0.14	1.93	31.04	231.83	<0.0001	46.41	<0.0001
**Total**										
PA-1	D^1.9378^	0.985	16.76	−1.36	12.46	11.11				
DV-1	D^1.5307^	0.985	16.86	−0.23	12.70	11.18	17.47	0.0007	0.82	0.5168
PA-2	D^1.5013^	0.988	15.01	−0.79	10.74	10.01				
DV-2	D^1.3057^	0.989	15.58	−0.81	11.14	10.33	11.10	0.0299	0.32	0.9239

*R*^2^: the coefficient of determination; RMSE: root mean square error; ME: mean error; MAE: mean absolute error; MAPE: mean absolute percentage error; Pr > |*L*|: the P value of the *L*-test; Pr > |*F*|: the *p* value of the *F*-test.
